# Functional subgroups of cochlear inner hair cell ribbon synapses differently modulate their EPSC properties in response to stimulation

**DOI:** 10.1152/jn.00452.2020

**Published:** 2021-05-05

**Authors:** Mamiko Niwa, Eric D. Young, Elisabeth Glowatzki, Anthony J. Ricci

**Affiliations:** ^1^Department of Otolaryngology–Head and Neck Surgery, Stanford University School of Medicine, Stanford, California; ^2^Center for Hearing and Balance, Johns Hopkins School of Medicine, Baltimore, Maryland; ^3^Department of Biomedical Engineering, Johns Hopkins School of Medicine, Baltimore, Maryland; ^4^Department of Otolaryngology–Head, and Neck Surgery, Johns Hopkins School of Medicine, Baltimore, Maryland; ^5^Department of Neuroscience, Johns Hopkins School of Medicine, Baltimore, Maryland; ^6^Department of Molecular and Cellular Physiology, Stanford University, Stanford, California

**Keywords:** auditory, deconvolution, inner hair cell, spiral ganglion, synapses

## Abstract

Spiral ganglion neurons (SGNs) form single synapses on inner hair cells (IHCs), transforming sound-induced IHC receptor potentials into trains of action potentials. SGN neurons are classified by spontaneous firing rates as well as their threshold response to sound intensity levels. We investigated the hypothesis that synaptic specializations underlie mouse SGN response properties and vary with pillar versus modiloar synapse location around the hair cell. Depolarizing hair cells with 40 mM K^+^ increased the rate of postsynaptic responses. Pillar synapses matured later than modiolar synapses. Excitatory postsynaptic current (EPSC) amplitude, area, and number of underlying events per EPSC were similar between synapse locations at steady state. However, modiolar synapses produced larger monophasic EPSCs when EPSC rates were low and EPSCs became more multiphasic and smaller in amplitude when rates were higher, while pillar synapses produced more monophasic and larger EPSCs when the release rates were higher. We propose that pillar and modiolar synapses have different operating points. Our data provide insight into underlying mechanisms regulating EPSC generation.

**NEW & NOTEWORTHY** Data presented here provide the first direct functional evidence of late synaptic maturation of the hair cell- spiral ganglion neuron synapse, where pillar synapses mature after postnatal *day 20*. Data identify a presynaptic difference in release during stimulation. This difference may in part drive afferent firing properties.

## Graphical abstract

**Figure:**
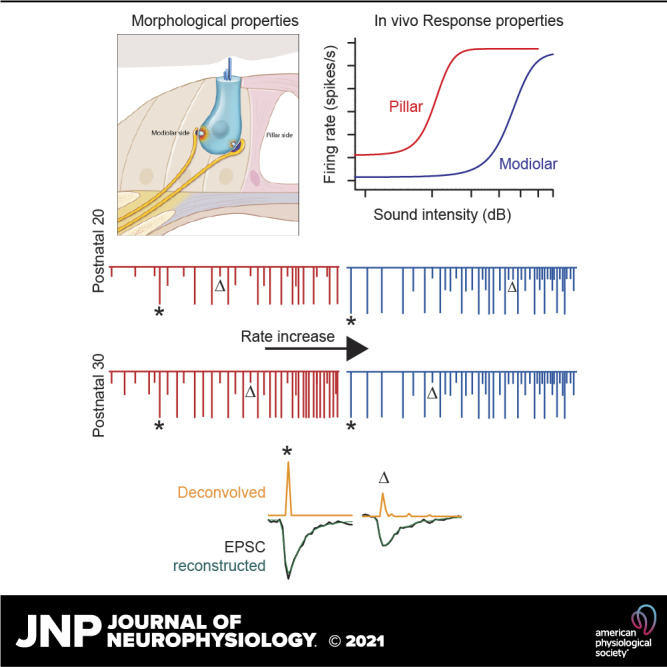

## INTRODUCTION

Mammalian cochlea synapses occur between inner hair cells (IHCs) and type I spiral ganglion neurons (SGNs) where a graded hair cell receptor potential is transformed into a train of action potentials in SGNs. Type-I SGNs form single synapses with hair cells and are heterogeneous in firing properties, often categorized based on spontaneous firing rates (SRs) (1–3). Recently, genomic data identified at least three subclasses of SGNs differing in ion channel, ryanodine receptor, and calcium binding protein expression, hypothesized to correlate with the physiologically identified subgroups (4–6). High-SR SGNs detect lower intensity sounds and show response saturation to mid-to-high intensity tones, while low-SR fibers have higher intensity thresholds with a nonsaturating response to high intensity tones (2, 7). Heterogeneity in the SR of SGNs is consistent across species suggesting very foundational mechanisms for information transfer [cats (2, 8); gerbils (9); rats (10); mice (11); and chinchillas (12)]. SGN SR correlates with dynamic range (2, 7, 12). High-SR fibers are less sensitive to amplitude modulated sounds (8), have a greater magnitude of onset response, have faster rates of adaptation (13), and are more resistant to masking noise (14) compared with low-SR fibers. These groups of fibers also differ in their projection patterns to the cochlear nucleus (15, 16). Thus SGN subgroups classified by SR likely make distinct contributions to auditory functions. Despite the physiological importance of the distinct response properties of SGN subgroups, it is not fully understood how/when individual fibers acquire their specific response characteristics during development and whether the underlying mechanisms arise pre- and/or postsynaptically.

**Figure 2.**
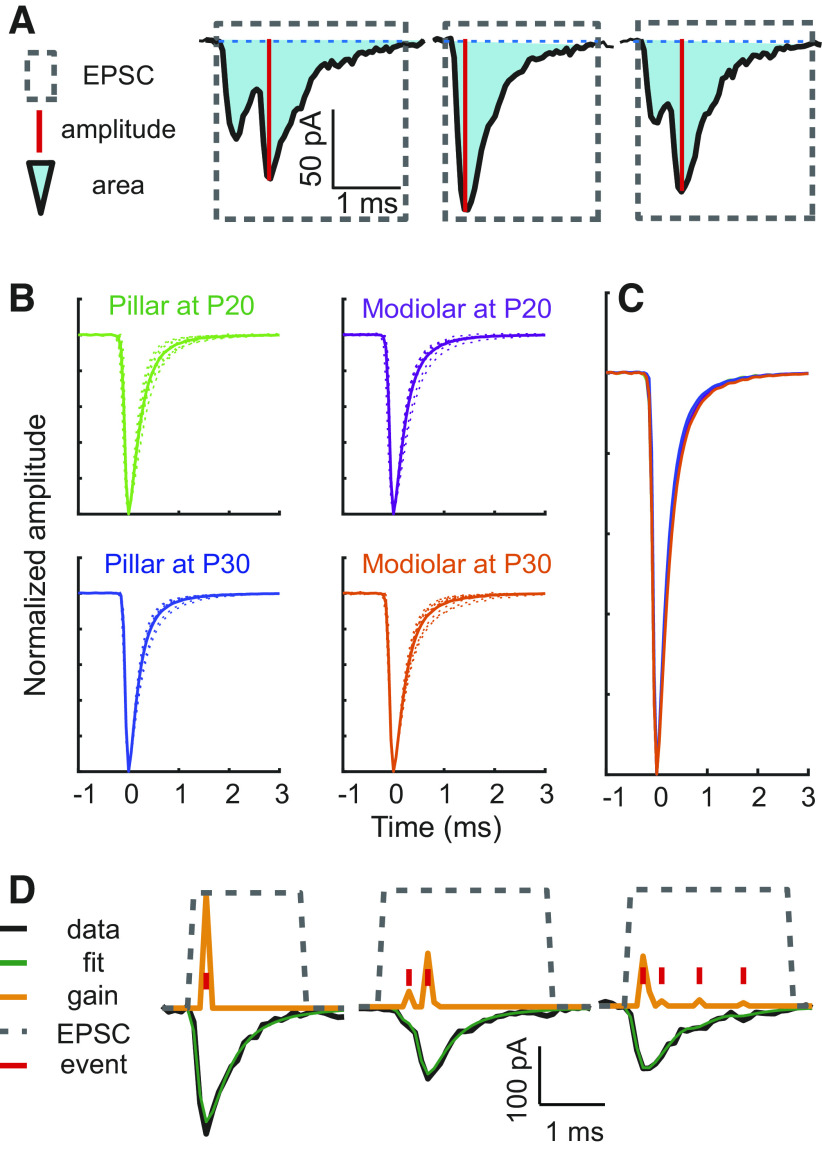
Excitatory postsynaptic currents (EPSCs) examples and definitions of variables used in the deconvolution analysis. *A*: zoomed-in view of the current trace showing three EPSC examples from Fig. 1. Our custom-developed, deconvolution program defines EPSCs as the regions of current trace deflected below an empirically determined threshold level. EPSC boundaries were set at 2% of the peak above baseline, denoted with dotted gray rectangles. EPSC amplitude is defined as the most negative point of the current trace measured from the baseline (solid red lines), and EPSC area is the integrated current over the entire width of the EPSC (light blue). *B*: waveforms of kernel, normalized average waveform of monophasic events, used in deconvolution for each synapse. In each panel, the dotted line denotes the kernel in an individual recording, and the solid line denotes the population average. P, postnatal day. *C*: population average waveforms of kernels from all 4 groups are overlaid to show the similarity between groups. *D*: 3 EPSCs were deconvolved to 1 (*left*), 2 (*middle*), and 4 (*right*) comprising events. The EPSC with 1 event (*left*) has the waveform like the kernel used in the deconvolution. Red ticks indicate the timing of each event, and the yellow lines indicate its gain (scaling coefficient). Blue trace is the original current measurement, and the green trace is the fit of the deconvolution result (the linear summation of the kernel scaled by event gains).

The anatomical location of synapses is defined as proximity to the central modiolus, where SGN fibers are bundled into the eighth nerve or to the pillar cells that create the tunnel of Corti (see Fig. 2*B*). High-SR fibers tend to make synaptic contacts on the pillar side of IHCs whereas low-SR fibers contact the modiolar side (17). Presynaptically, the number of Ca^2+^ channels inferred by immunohistochemistry and Ca^2+^ current influx is larger in modiolar-side compared with pillar-side presynaptic terminals (18, 19). Also, presynaptic electron-dense ribbon structures are smaller at pillar-side presynaptic terminals (20). Postsynaptically, pillar-side boutons have bigger glutamate receptor patches compared with the modiolar side (20). Recent data suggest that high thresholds to injected current correspond with high threshold sound detection (21). Thus both, pre- and postsynaptic mechanisms likely contribute to establishing the SR and dynamic range of SGNs.

We focused on identifying synaptic differences between pillar and modiolar synapses at postnatal days (P) 20 and 30, ages suggested to encompass synaptic maturation (20, 22). Excitatory postsynaptic currents (EPSCs) from SGN terminal endings were recorded, and synaptic activity was increased by perfusing 40 mM external potassium. We find *1*) properties of pillar fibers but not modiolar fibers continue to mature between P20 and P30, and *2*) EPSC amplitude, charge, and waveform change dynamically with EPSC rate, and the dynamics differ between fibers located on pillar and modiolar sides. These differences likely reflect differences in biological mechanisms underlying the heterogeneity of SGNs between the two locations.

## METHODS

### Tissue Preparation

The Administrative Panel on Laboratory Animal Care at Stanford University (APLAC No. 14345) approved all animal procedures. C57bl6 mice of both sexes at ages P17 to P36 were used. Mice were anesthetized with isoflurane and decapitated. The cochlea was acutely dissected from the temporal bone. After the bone was chipped off and the stria vascularis was removed, the apical coil of the cochlea was excised with the spiral ganglion left intact as previously described (23). We recorded from apical regions in the 2- to 4-kHz range. The tectorial membrane was manually removed. Excess bone at the center of the apical coil was carefully removed so that the tissue was less bulky and would lie flat. Dissection was done in chilled (∼4°C) standard extracellular solution containing 1 mM curare to block mechanosensitive ion channels (24, 25). Then, the excised tissue was placed under two dental floss fibers in a recording chamber filled with the standard extracellular solution at room temperature (20–22°C). The nonphysiological temperature likely reduces the calcium current and so overall reduces release rates; in our hands the tissue degrades at a rate making comparisons at the older ages less reliable. As we are comparing between synapse populations, we are assuming comparable effects of temperature on both populations of synapses. The standard external solution contained the following (in mM): 5.8 KCl, 155 NaCl, 0.9 MgCl_2_, 1.3 CaCl_2_, 0.7 NaH_2_PO_4_, 5.6 d-glucose, 10 HEPES, 2 Na-ascorbate, 2 Na-pyruvate, 2 creatine monohydrate, and ∼305 mosmol/H_2_O, pH 7.4 (NaOH).

### Bouton Identification

**Figure 5.**
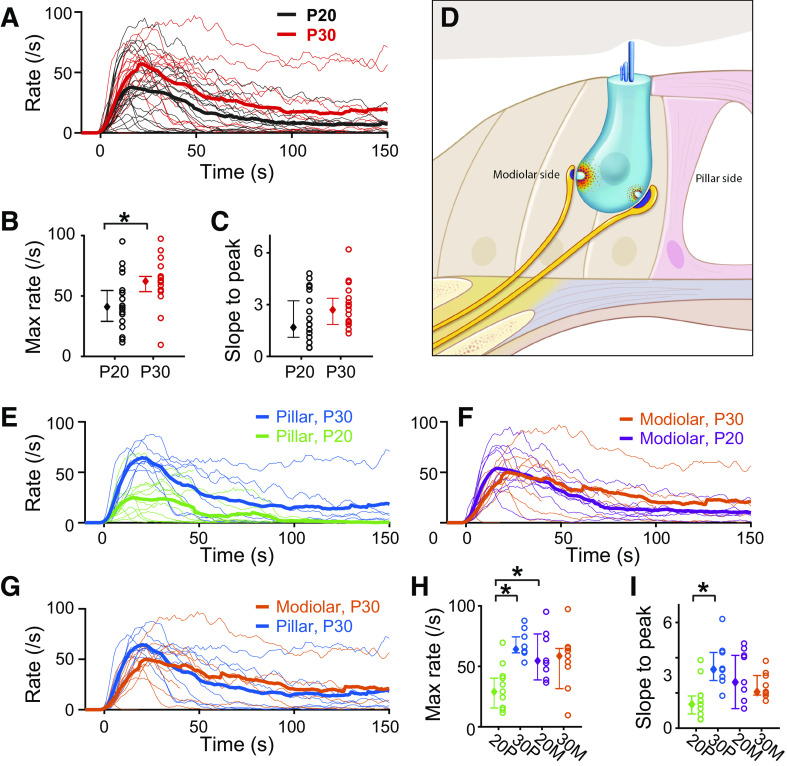
Excitatory postsynaptic currents (EPSC) rates vary with age and synapse position. *A*: plot of EPSC rate vs. time for all synapses that showed significant response to 40-mM K^+^ application. The population mean EPSC rate is shown in a thick trace for postnatal day (P) 20 animals (black, *n* = 20) and for P30 (red, *n *=* *17). Individual fibers are shown in thin traces of corresponding colors. Pillar and modiolar-side fibers are combined. The start of the response to 40-mM K^+^ application is defined as the first-time bin where the EPSC rate exceeded the 0 EPSCs/s (baseline rate in 5.8 mM K^+^) for 2 consecutive bins, and the start time is aligned at time = 0 s for all recordings. *B*: maximal EPSC rate, defined as the highest rate reached after 40-mM K^+^ application, is compared between P20 and P30 fibers and shows a significant difference by rank-sum test (*P* = 0.0293). Open circles denote the values of individual fibers, and the closed diamond is the median value. The error bars are 25 and 75 percentile values (the same applies for *C*, *G*, and *H*). *C*: the slope to peak, defined as the maximal EPSC rate divided by the time to reach the peak from the start of the response, is not significantly different between P20 and 30 (*P* = 0.0797 by rank-sum test). *D*: a schematized inner hair cell with the 2 morphologically defined population of synapses. Additionally, the red cloud depicts differences in calcium channel densities, the light blue represents the ribbon structure, and the dark blue postsynaptically represents identified differences in glutamate receptor distributions. *E* and *F*: plots of EPSC rate vs. time are shown separately for pillar (*E*) and modiolar (*F*) locations; *n* = 9 for pillar fibers at P20 (green), *n* = 8 for pillar fibers at P30 (blue), *n* = 11 for modiolar fibers at P20 (purple), and *n* = 9 for modiolar fibers at P30 (orange). *G*: plots of EPSC rate vs. time are compared between pillar vs. modiolar fibers at P30. *H*: the maximal EPSC rate is compared among 4 groups: 20 P, pillar at P20; 30 P, pillar at P30; 20 M, modiolar at P20; and 30 M, modiolar at P30. Maximum rate significantly increased with maturation in pillar nerves from P20 to P30 (*P* = 0.0017 by two-way ANOVA) but not in modiolar nerves. At P20, the maximal EPSC rate is significantly greater for modiolar synapses than pillar. *I*: slope to peak increased significantly with maturation in pillar synapses from P20 to P30 (*P* = 0.0028 by two-way ANOVA) but not in modiolar synapses. The colors used to identify age and synapse location are continued through all figures. *Statistical significance for the compared measurements.

We identified pillar and modiolar fibers at the time of each recording based on their location along the basolateral surface of the IHC (see schematic in Fig. 5*D*). Since the hair cells in the excised cochlea lie flat at the animal ages we used for testing, modiolar fibers’ boutons are located near the surface of the preparation, while pillar fibers are located deeper in the tissue, passing underneath the hair cells’ nucleus. Pillar side boutons were identified and approached by first removing the pillar cells and bringing the recording electrode into the tissue through that vacated space as a means of accessing boutons and not the hair cell body. Modiolar fibers were accessed by first generating a hole near the basolateral side of the IHC and bringing the recording electrode down where the boutons were located on the upper face of the hair cell. We tried not to sample from boutons that were at ambiguous locations. These two approaches ensured that the bouton was located between the recording pipette and the hair cell soma.

### Recording Conditions

**Figure 1.**
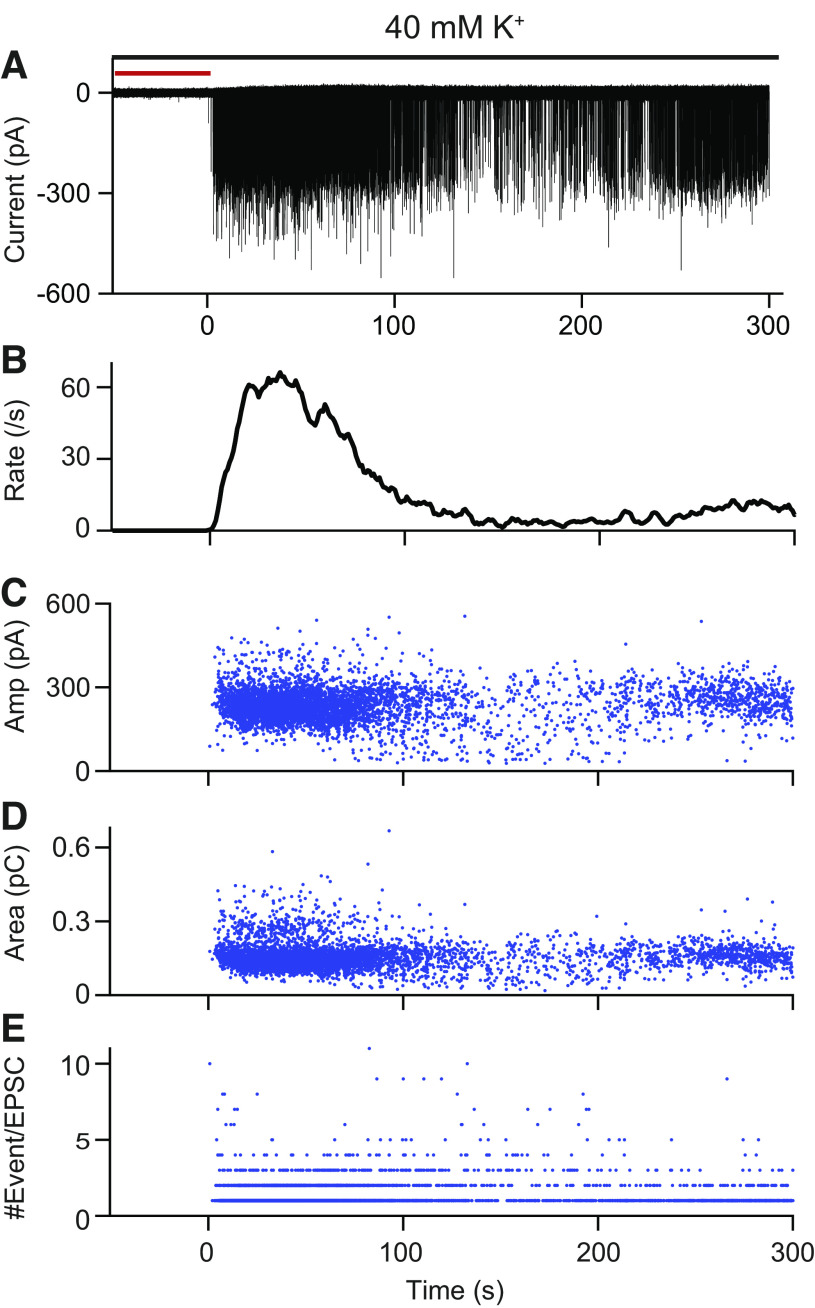
Example of postsynaptic recording in response to potassium application with 1st level summary of analysis used throughout the article. *A*: whole cell, voltage-clamp recording from a postntal *day 36* pillar terminal ending; 40 mM K^+^ were applied onto the organ of Corti for the entire period shown as indicated by the black bar. The red bar indicates the preresponse time where perfusion has begun but the increased release rate has not. The start of the synaptic response is aligned at time = 0 s. *B*: excitatory postsynaptic current (EPSC) rate of the synapse shown in *A*. *C*–*E*: amplitude (*C*), area (*D*), and number of events (*E*) per EPSC are plotted over time for the recording shown in *A*. The number of events comprising each EPSC was determined by deconvolution. Each EPSC is denoted by a blue dot.

Recording pipettes were made from 1.5-mm, thick-walled borosilicate glass (WPI). Pipettes were pulled using a multistep horizontal puller (Sutter) and fire polished while pressure was applied from the back end of the pipettes to reduce resistance to 6–8 MΩ (26). The use of pressure enabled us to reduce pipette resistance by ∼5 MΩ, compared with fire polishing them without pressure to a similar size of tip opening. A recording pipette filled with internal solution (in mM): 135 KCl, 3.5 MgCl_2_, 0.1 CaCl_2_, 5.0 EGTA, 5.0 HEPES, 2.5 Na_2_-ATP, pH 7.2 KOH, and ∼290 mosmol/H_2_O was directed at ∼45° against the row of inner hair cells while applying positive pressure so that the pipette tip remained free of debris and created a liquid-filled space between inner hair cells and supporting cells, where the nerve endings attach to IHCs (23). The pressure was kept at a minimum so that the high K^+^ -concentration in the internal solution did not overstimulate the terminal endings. When a whole cell, voltage-clamp recording was achieved from a terminal ending (27), 5 µM tetrodotoxin (Sigma-Aldrich) were perfused into the chamber to block sodium channels. Membrane currents were recorded in the standard external solution (with 5.8 mM K^+^, see above) at room temperature for at least 3 min to establish the rate of spontaneously occurring EPSCs. Then, 40 mM K^+^ solution (K^+^ was equimolar substituted for Na^+^ in the standard external solution) was applied to depolarize IHCs and induce synaptic vesicle release, and the membrane current was recorded for 1∼5 min, depending on the duration of synaptic activity in each afferent fiber (Fig. 1) (23). The terminal endings were voltage-clamped to a membrane potential of −85 mV. The series resistance was un-compensated (40.0 ± 11.2 MΩ, *n* = 40). A Cambridge Electronic Design (CED) system (model 1401) was used to digitize currents, the patch amplifier was driven using JClamp software (SciSoft). Data were digitized at 20 kHz for offline analysis.

### Data Analysis

Data presented are from 40 terminal recordings, each of which came from a different mouse (*n* = 11 for P20-pillar group; *n* = 11 for P20-modiolar group; *n* = 9 for P30-pillar group; and *n* = 9 for P30-modiolar group). From these, two recordings had EPSCs in the standard extracellular solution (5.8 mM K^+^) but did not increase in rate when switched from 5.8 to 40 mM K^ +^. These were excluded from any analysis that required the time of response start but were included for the analysis of EPSC amplitude and area across the entire recording time. Another recording, which showed only a very brief response to 40 mM K^+^ (total 22 EPSCs evoked in <2-s duration), was excluded from the analysis.

Matlab R2015b and R2017a were used for all data and statistical analyses. A custom program was developed for EPSC identification and deconvolution of EPSC waveforms, and the procedures are fully described in the companion paper Young et al. (28). Matlab built-in function ranksum() was used for nonparametric comparisons between two age groups (Wilcoxon rank sum test). The function anovan() was used for two-way ANOVA unbalanced design, where age and location of fibers were treated as the two factors. The function multcompare() was used to test which age-location groups are significantly different from each other, where Tukey’s honestly significant difference procedure was used to set the type of critical value.

Figure 1 introduces the time course and data analysis for the experiments. The plot of SGN K^+^ evoked currents in Fig. 1*A* shows robust synaptic activity recorded from a pillar terminal of a P36 animal. The extracellular solution is switched from 5.8 to 40 mM K^+^ solution at the start of the plot through a local apical perfusion pipette and is applied for the entire duration of the plot. There is a delay between switching on 40 mM K^+^ and when it reaches the tissue. During this delay, the fiber showed few EPSCs (see red line in Fig. 1*A*). The point at which the rate increases in response to a 40-mM K^+^ application is set as time 0 in all figures. During the 40-mM K^+^ application, the EPSC rate increased to a maximal EPSC rate of ∼60 s^−1^, and then gradually, over about two minutes declined to a lower rate of <10 s^−1^ and stayed at that rate for another 2 min (Fig. 1*B*).

### EPSC Identification

First, the current record baseline was set to zero. EPSCs were estimated as regions in the current signal where the membrane current deflected more negative than an empirically chosen threshold. A threshold was chosen by going through the current signal trace so that small EPSCs would not be missed. Then, the median of non-EPSC points was calculated in overlapping 10-ms windows. The computed baseline was linearly interpolated to cover the regions of EPSCs (to the sampling rate) and subtracted from the entire current record. After the baseline subtraction, EPSCs were redefined with the same threshold above. To ensure that small EPSCs were included in the data with the chosen threshold, the program displayed the waveforms of the currents that would have been triggered by lower threshold settings. Users can either *1*) adjust the threshold lower and redefine the EPSCs, or *2*) add these small waveforms to the data set of EPSCs if they confirmed these waveforms as EPSCs by visual inspection. The end of the EPSC was defined by the current returning to baseline.

With EPSCs defined, their amplitude, area, and frequency were calculated (Fig. 1, *B–D*; Fig. 2*A*). The EPSC amplitude, EPSC area, and the number of events comprising each EPSC are plotted in Fig. 1, *C–E*, respectively, for the current shown in Fig. 1*A*. EPSC amplitude was defined as the most negative value within the EPSC waveform (with the baseline set to zero, Fig. 2*A*). EPSC area was computed by integrating the EPSC waveform over time, thus representing the charge carried by each EPSC. Rate was calculated by counting EPSCs in a 1-s window for the 40-mM K^+^ conditions (23). Each blue dot denotes an EPSC. The start of the response to the 40-mM K^+^ application was defined as the first time bin where the EPSC rate exceeded the 0 Hz (baseline rate in 5.8 mM K^+^) for two consecutive bins. Each variable has a large spread at any given time point and changes over the time course of the 40-mM K^+^ application. In the following figures, we present how the EPSC rate, amplitude, area, and number of events/EPSC change with age and locations of nerve terminals and how these variables interact with each other.

Three segments of the expanded current trace in Fig. 1 are presented in Fig. 2*A* showing that EPSCs have variable shapes. Some are monophasic, with a single peak and exponential decay (Fig. 2*A*, *middle*), while others are multiphasic (Fig. 2*A*, *left* and *right*), with more than one peak, as if there are multiple, smaller events summating to the final, complex waveform. The EPSCs are defined as the current from the time it moves from the baseline until the time it returns to baseline. We can quantify the EPSC’s properties with measurements such as amplitude (denoted by red line in Fig. 2*A*) as the largest current deflection from baseline and area (denoted by pale blue shade) as the integral of the current for the entire duration of the EPSC.

### Deconvolution

Each EPSC waveform was deconvolved to determine the number and timing of comprising events [Young et al. (28)], which gave us a means of quantifying the complexity of waveforms in more details. We assume that there is an underlying discrete event, whose waveform is defined as the “kernel” and that the waveform of an EPSC is a linear sum of multiple kernels scaled by independent coefficients. We make no assumptions as to the underlying mechanism generating this kernel. Therefore, the waveform of an EPSC, *ê*(*t*), can be estimated by:

(*1*) $$\hat{e}(t)=\sum_{j=1}^{j}a_{j}k(t-t_{j})$$

where *k*(*t*) is the kernel, and scaling coefficient *a_j_* and occurrence times *t_j_* for *j*-th event, and the total number of events *J*. We assumed that the waveform of monophasic EPSCs is the kernel, because we found that most monophasic waveforms are scaled versions of the same waveform [Fig. 2, *B* and *C*; Young et al. (28)] To isolate monophasic EPSCs, all EPSCs were normalized to a peak amplitude of −1 and superimposed on each other by aligning them at the largest negative peaks on an interactive display. Then, multiphasic waveforms were manually eliminated from the display by setting limits on the widths of waveforms. The average of monophasic EPSCs isolated as such was used as an estimate of the kernel. Only EPSCs that were preceded and followed by baseline for at least 10 ms were shown on the display; these were used to estimate the kernel. The kernel was defined separately for each fiber (Fig. 2*B*). Examples of kernels from all the fibers used here are presented in Fig. 2, *B* and *C*, where the similarity between kernels across age and location is apparent. We also compared the decay time of EPSCs throughout the recording and found no time-dependent changes, suggesting a common kernel for each recording.

The deconvolution model determined the total number of events *J*, a set of event areas {*a_j_*}, and a set of event times for each EPSC by minimizing the error defined as:

(*2*) $$E=\sum_{r=r_{3}}^{r_{1}}{\left(\hat{e}(t)-e(t)\right)}^{2}+\lambda \sum_{i=1}^{j}\left|a_{i}\right|.$$

The first term in the error, *E*, is the squared differences between the actual EPSC waveform, *e*(*t*), and the model fit, *ê*(*t*), given by *Eq. 1* [see companion paper Young et al. (28)] This error can almost always be reduced by adding more small kernels; however, the addition of more kernels after some values of *J* is likely fitting the noise in the data. Thus the second Σ term in the error E is placed to deal with this overfitting by penalizing the addition of more kernels. This method is called lasso [Tibshirani (29)], and allows the model to add another kernel into *ê*(*t*) only if the squared error reduced by the addition is greater than the penalty. The hyperparameter λ decides the magnitude of penalty. It was set to the value where the squared error exceeds its minimum by 1 SE (estimated by 5-fold cross validation; Young et al. (28)].

### Removing “Doubles”

**Figure 3.**
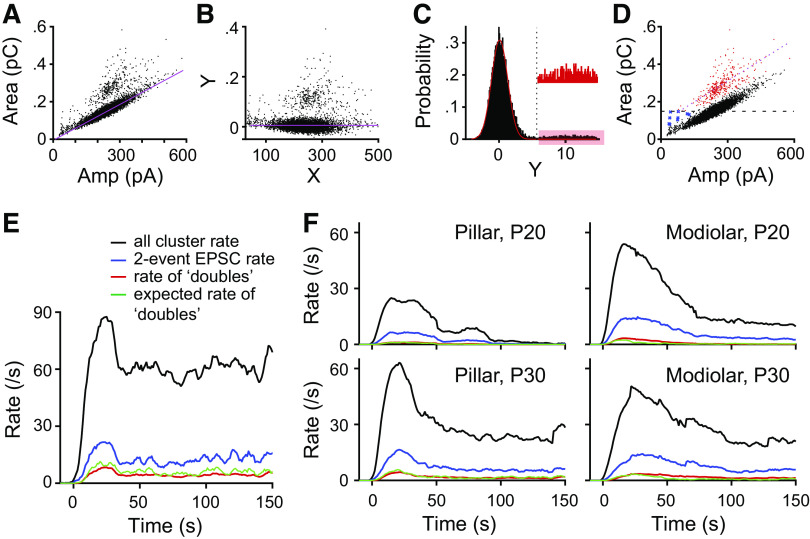
Method for removal of events representing simultaneous release from multiple sites (doubles). At high release rates, simultaneous release from independent release sites, termed doubles contaminate analysis and so were removed using the following analysis. *A*: plot of excitatory postsynaptic current (EPSC) area against EPSC amplitude for a postnatal day (P) 36 pillar fiber. Each black dot denotes an EPSC. The magenta line shows the linear fit on the plot of EPSC area vs. amplitude including only monophasic EPSCs (#event = 1 by deconvolution). *B*: the plot in *A* was shifted by the *y*-intercept of the linear fit in *y*-direction and rotated around the origin so that the linear fit lies on the *x*-axis. *C*: probability histogram of *Y* values of *D* shows 2 distributions; major 1 centered around *Y* = 0. The dotted black line denotes the cutoff (the means + 4 × SD of the major distribution) used to divide EPSCs into 2 distributions. The *inset* scales the shaded region that represents the second distribution of EPSCs. *D*: the same plot as in *A*, but those EPSCs isolated using the cutoff in *C* are shown in red dots and blue crosses. Blue crosses have areas below the mean area of monophasic EPSCs (shown by black dotted line), which argues against them being “doubles”; therefore, only red dots were labeled as “doubles.” *E*: plots of EPSC rate vs. time for all EPSCs (black) for the response of the synapse shown in *A*–*D*. The blue trace shows the rate only counting EPSCs with #event = 2 from deconvolution. The red trace shows the rate only counting those EPSCs depicted in red in *D*. The green trace shows the expected rate of observing two independent EPSCs temporally overwrapped based on the ongoing EPSC rate (black). *F*: population average EPSC rate vs. time for P20 pillar (*top left*), P20 modiolar (*top right*), P30 pillar (*bottom left*), and P30 modiolar (*bottom right*). The color schemes are the same as in *E*.

**Figure 12.**
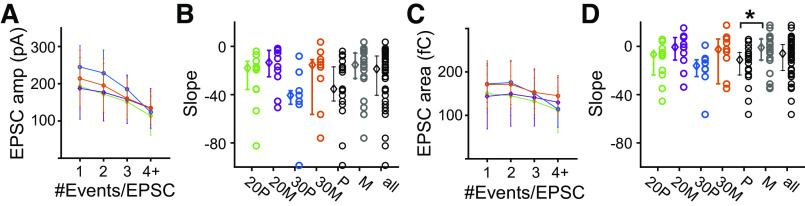
*A*: excitatory postsynaptic current (EPSC) amplitude decreases as #events/EPSC increases. Population-averaged median EPSC amplitude as a function of #events/EPSC for postnatal day (P) 20 pillar (20P; green), P20 modiolar (20M; purple), P30 pillar (30P; blue), and P30 modiolar (30M; orange). The error bar is SD. *B*: the steepest slope of the EPSC amplitude vs #events/EPSC relationship is presented for each fiber and compared among the 4 groups. The slope was obtained from a linear regression to the part of the curve containing 2 or more events/EPSC. Open circles denote the values of individual fibers, and the closed diamond is the median value. The error bars are 25 and 75 percentile values. *C* and *D*: same as *A* and *B* but for EPSC area. Modiolar synapses had a steeper slope than pillar synapses (*P* = 0.045). *Statistical significance for the compared measurements.

A separate population of events is observed when rates are elevated. An example of this population is shown in Fig. 1*D*, where there is a second cluster of spikes during the first 50 s, having an area close to double the mean for the entire measurement. This is likely due to temporal overlap between independent release sites. We developed an approach to identify and remove overlapping EPSCs, termed “doubles” from the datasets to determine whether their presence altered the overall responses (Fig. 3). Figure 3*A* plots amplitude against area with each dot representing a monophasic EPSC. A linear regression (denoted by a magenta line in Fig. 3*A*) was done on the subset of data only including monophasic EPSCs to obtain a slope and *y*-intercept. Then, data from Fig. 3*A* are shifted by the *x*-axis intercept and rotated around the origin by the slope of the fit (Fig. 3*B*). The probability histogram in Fig. 3*C* shows two peaks where the initial peak is centered around 0 and the second peak is broad and centered around 10 (see Fig. 3*C*, *inset*). The red line on Fig. 3*C* shows the single-Gaussian fit on the probability distribution, from which the means ± SD were obtained. The dotted line in the plot illustrates the cut-off (four times the SD) used to separate the two distributions. Figure 3*D* replots the data from Fig. 3*A* now including distribution data where the red dots and blue crosses represent EPSCs whose values are greater than dotted line from Fig. 3*C*. Only red dots are considered doubles because they are actually larger than the mean monophasic area; however, the blue crosses have smaller means and so are unlikely doubles (kept in the data pool as nondoubles). What these EPSCs represent remains to be determined. Doubles are expected to occur in a release rate dependent manner so that as release probability increases so too would the predicted rate of doubles. Figure 3, *E* and *F*, illustrates these predictions for the exemplar response of Fig. 3*A* (Fig. 3*E*) as well as summarized for age and location (Fig. 3*F*). No difference was observed for age or location in prevalence of doubles except that doubles are more prevalent in fibers having higher EPSC rates. In addition, expected rate of doubles was very similar to the observed rate of doubles isolated in the manner described above. Doubles were removed from data presented in Fig. 12 to ensure there was no inadvertent bias. All other figures include all events. The effect of doubles was evaluated on all data sets and found to not alter the fundamental conclusions and so were retained.

## RESULTS

**Figure 6.**
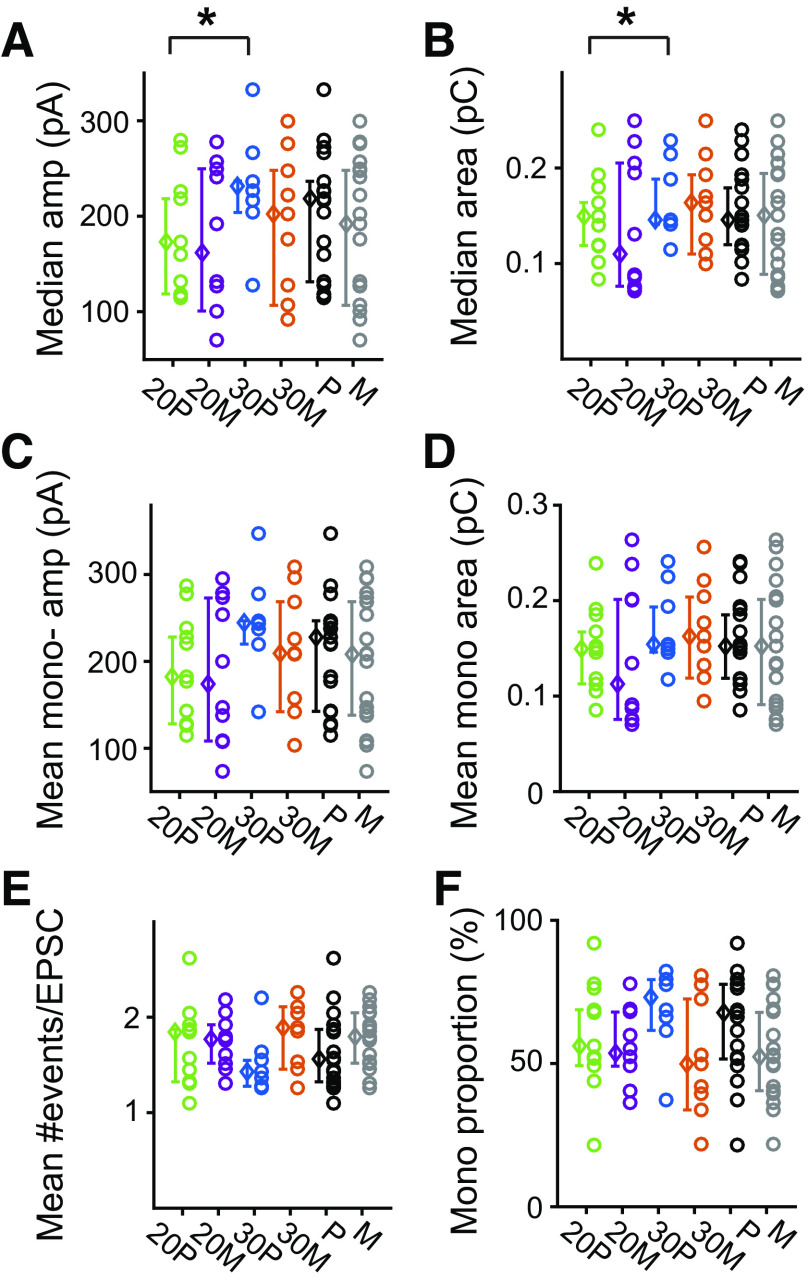
Excitatory postsynaptic currents (EPSC) properties across the entire recording vary with age but not between synaptic location during elevated rates. Median amplitude (*A*) and area (*B*) are presented for the total duration of K^+^ stimulation. Individual fibers are shown as open circles, color coded for age and synapse location: 20 P, pillar at postnatal day (P) 20; 30 P, pillar at P30; 20 M, modiolar at P20; and 30 M, modiolar at P30. The average ± SD for each synapses group is shown to the *left* as open diamonds. Pillar side synapses showed an increase in amplitude and area between postnatal day (P) 20 and P30 (*P* = 0.0068 and 0.0241, respectively), but no difference was observed between synapse location at either age. Monophasic EPSC amplitude (*C*) and area (*D*) showed similar trends as total EPSC population but did not reach statistical significance. No difference in either the #events per EPSC (*E*) nor in the proportion of monophasic events (*F*) was observed between ages or synaptic locations. *Statistical significance for the compared measurements.

Recordings from SGNs innervating inner hair cells (IHCs) were done to identify differences in their synaptic response based on age and synapse location. Whole cell, voltage-clamp recordings were obtained from bouton endings of single SGNs directly where they contact the IHC and EPSC rate and waveform properties were characterized. Voltage clamping these neurons controlled for any postsynaptic electrical differences such as input resistance or complement of ion channels, allowing us to focus specifically in synaptic differences, even during the application of a high potassium solution. Recordings were performed in C57BL6 mice of either sex at two ages; P20 (ranged from P17 to 20, mean = P18.7 ± 0.97, *n* = 22) and P30 (ranged from P28 to 36, mean = P32.2± 3.2, *n* = 18). These ages were chosen because SGNs continue to mature after the onset of hearing (∼2 wk postnatally), as shown by the changes in the distribution of spontaneous rates for fibers of these two age ranges (22) and histochemically by changes in synaptic ribbon size and postsynaptic glutamate receptor area (see cartoon in Fig. 6*D*) (20). Secondly, recordings were made from bouton endings located on either the pillar or modiolar side of IHCs to compare properties between morphologically and functionally distinct fiber populations (see diagram in Fig. 6*D*). To capture possible changes in EPSC rate and waveform during hair cell depolarization, a 40-mM K^+^ solution was applied to induce hair cell depolarization. Previous work demonstrated that this treatment depolarized hair cells to ∼-25 mV within a few seconds and the hair cell membrane potential remained stable throughout the application (23).

The recorded fibers (∼90%) showed low to no EPSC activity in the standard physiological solution containing 5.8 mM K^+^ at room temperature. A previous study investigating activity in rat SGN endings performed under similar conditions, reported spontaneous firing by loose-patch extracellular and EPSC rates in the range of 2–67/s (22). When we recorded from a small number of IHCs prepared in the same manner as for terminal recordings, we noted that their resting potentials were often more hyperpolarized than −65 mV, a potential that would reduce synaptic activity because of the limited calcium conductance (23, 30). Thus EPSCs were evoked by depolarizing IHCs with a 40-mM K^+^ solution after the recording was established in a 5.8-mM K^+^ solution.

**Figure 4.**
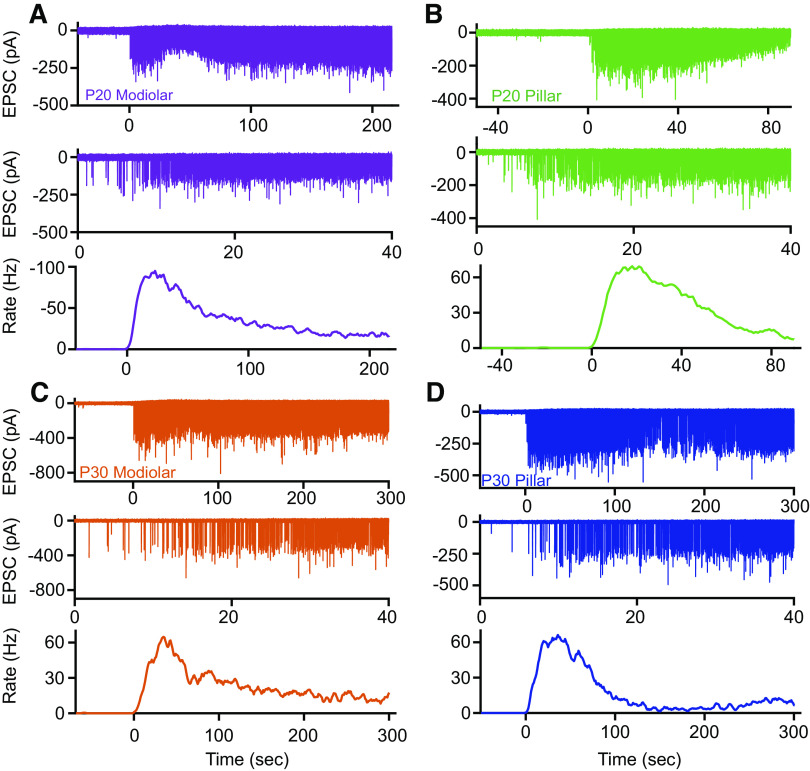
Examples of postsynaptic recordings made at postnatal *days 20* and *30* (P20 and P30) in response to 40-mM K^+^ application. *A*–*D*, *top*: long-term response of postsynaptic fibers where *time 0* represents the onset of the K^+^ response from P20 modiolar (*A*), P20 pillar (*B*), P30 modiolar (*C*), and P30 pillar (*D*) side synapse. *Time 0* is set as the time where excitatory postsynaptic currents (EPSC) rate first increases. The color code shown for each fiber type is maintained throughout the article. *A*–*D*, *middle*: expansion of the first 40 s of the response from *time 0*. The expansion better illustrates the initial amplitude differences between synapse types and ages. *A*–*D*, *bottom*: change in EPSC rate for the example presented above.

Examples of modiolar and pillar bouton recordings are presented in Fig. 4 as representative traces. *Time 0* represents the onset of response to elevated potassium. These data show qualitatively that EPSC rates are very low before 40-mM K^+^ application and increase quickly following application of 40 mM K^ +^. An expanded time view is presented for the onset response as it represents a key region selected for further analysis. During the initial increase in activity a change in EPSC amplitude is observed for pillar but not modiolar fibers (quantified below). At each age the rate of EPSCs increases rapidly and then decreases to a baseline rate for the remainder of the stimulus period.

In this study, we first analyze fundamental properties of EPSCs including rate, amplitude, and area for the entire stimulation period comparing both age and location. We also use a deconvolution algorithm to explore the underlying composition of these EPSCs. We next investigate these same properties in the time window where rate is increasing to its peak and again as the rate decreases to its steady state to better capture changes in the release properties during dynamical changes. Finally, we use the deconvolution algorithm to explore intra-EPSC dynamics to gain insight into potential mechanisms generating EPSCs and whether these might vary with age or synaptic location.

### Maximal EPSC Rate Increases with Maturation from P20 to P30

Figure 5*A* compares how the 40 mM K^+^-induced EPSC rate changes with age, between fibers of P20 animals (*n* = 20) and P30 animals (*n* = 17). The maximal EPSC rate at P30 is significantly greater than at P20 (*P* = 0.0293 by rank sum test, Fig. 5*B*). In contrast, the steady-state rate, measured at 90 s after the start of the response to 40 mM K^+^, does not significantly increase with age (*P* = 0.181 by rank sum test); however, the large variance in the rates among fibers may be responsible for the lack of significance. Figure 5*A* demonstrates a highly variable EPSC rate between fibers after the response onset. High variability is observed among fibers of the same age group as well. In addition, we did not find a significant difference in the slope to the maximal response (maximal EPSC rate divided by the time it took from the start of the response to the peak, Fig. 5*C*, *P* = 0.08 by rank sum test) nor in the ratio of steady state to maximal EPSC rate (*P* = 0.250 by rank sum test; data not shown) between the two age groups, though the trend is like the maximal rate measurement. These data suggest that presynaptic changes occur between P20 and P30 that enhance the ability of the synapse to operate at higher rates.

### The Increase in the Maximal EPSC Rate from P20 to P30 Is Due to Developmental Changes Associated with Pillar Synapses

When we compare the change in EPSC rate with age at each location (Fig. 5*E* for pillar and Fig. 5*F* for modiolar), P30 pillar synapses have a significantly greater maximal EPSC rate (*P* = 0.0021 by two-way ANOVA, Fig. 5*H*) and a steeper slope to peak (*P* = 0.0032 by two-way ANOVA, Fig. 5*I*) compared with P20 pillar synapses. In contrast, P30 modiolar synapses do not show any significant difference in maximal EPSC rate compared with P20 modiolar synapses (Fig. 5, *F* and *H*), nor a change in the slope to peak (Fig. 5*I*). Comparison of the EPSC rate between pillar versus modiolar synapses by age shows that at P20 the maximal EPSC rate is significantly lower in pillar compared with modiolar synapses but is statistically not different between the two locations at P30 (Fig. 5, *G* and *H*). In summary, on average changes between P20 and P30 occur to pillar but not to modiolar fibers. This is consistent with findings by Wu et al. (22) showing that at P30 a higher percentage of high spontaneous rate fibers (usually pillar fibers) were identified as compared with P20. Together, these studies suggest that in the P20/P30 time window maturation still occurs for pillar IHC/SGN synapses. It is surprising that maximal release rates were consistent between pillar and modiolar fibers in that high and low spontaneous rate fibers, potentially represented by these morphologically distinct groups, differ in maximal firing rates (2, 8). This difference may be species driven, a reflection of comparing in vivo to in vitro data or perhaps due to additional postsynaptic specializations.

### EPSC Amplitude, Area, and Number of Events/EPSC Are Similar between Synapse Location, While Pillar Synapses Have a Dependence on Age

We hypothesized that there would be differences in EPSC amplitudes or areas that correlate with differences in ribbon size, calcium channel density, and postsynaptic glutamate receptor plaque size as well as to account for differences in firing rates from pillar and modiolar fibers and those observed with age (20, 22). Surprisingly, we found no significant effect of location on the median amplitude or area (Fig. 6, *A* and *B*, *P* = 0.164 and *P* = 0.809, respectively, by two-way ANOVA). While modiolar synapses showed no significant difference in amplitude or area with age, pillar synapses showed an increase in both amplitude and area between P20 and P30 (*P* = 0.0068 and 0.0241, respectively).

When only the monophasic EPSCs (EPSCs with 1 event, see Fig. 2*A*) are analyzed, there was no significant effect of age or location on the mean monophasic EPSC amplitude (Fig. 6*C*, *P* = 0.164 and 0.317, respectively) or the mean monophasic EPSC area (Fig. 6*D*, *P* = 0.199 and 0.783, respectively). The deconvolution analysis also allowed us to determine the number of underlying events comprising each EPSC (where monophasic EPSC have 1 event and multiphasic EPSCs have more than 1 event see Fig. 2*D*). We found no significant effect of age or location on the mean number of events per EPSC (Fig. 6*E*, *P* = 0.094 and 0.417, respectively, by two-way ANOVA) or in the proportion of monophasic EPSCs within the total EPSC population of individual recordings (Fig. 6*F*, *P* = 0.612 and 0.077, respectively). Thus the underlying features of the synapses appear quite similar between location during prolonged stimulation, while pillar synapses continue to mature between P20 and P30.

### EPSC Amplitude, Area, and Number of Events Show Complex Dynamics When Evaluated from the Onset of 40-mM K^+^ Stimulus Until the Peak Rate Is Reached

**Figure 7.**
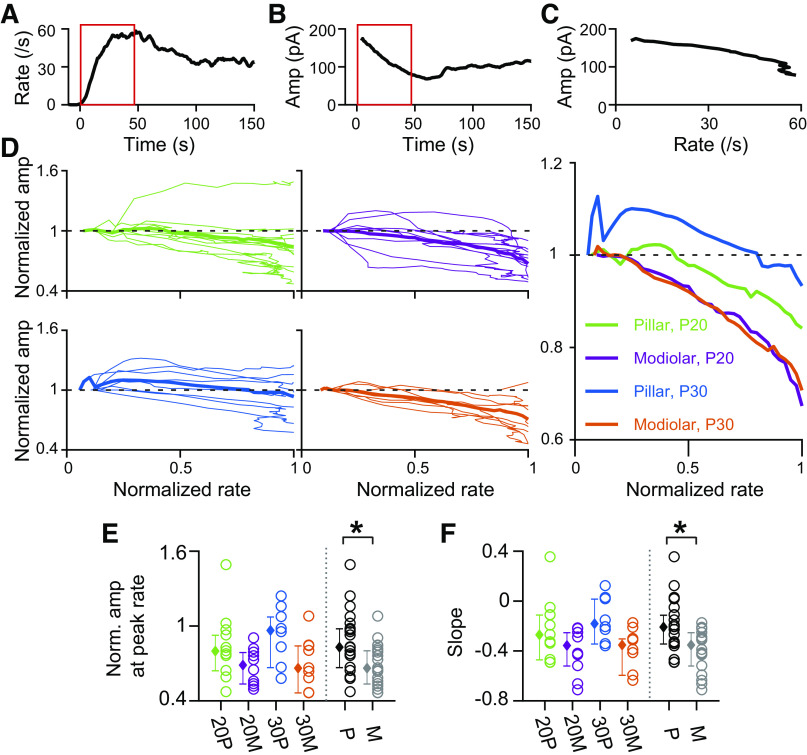
Excitatory postsynaptic current (EPSC) amplitude varies differently between pillar and modiolar side synapses as rate increases. *A* and *B*: plots of EPSC rate (*A*) and amplitude (*B*) over time for a postnatal day (P) 36 modiolar synapse, showing the first 150 s of response to 40-mM K^+^ application. The amplitude is calculated by averaging the amplitude of EPSCs in 1-s time intervals moving in 100-ms increments. The mean amplitude in *B* was calculated in all time bins that contained ≥5 EPSCs to reduce noise. The red rectangles denote the time window from the start of significant response to the peak response. This time period was used to analyze the dynamics of amplitude, area, and #events per EPSC in *A–F* as well as Figs. 8 and 9. *C*: plot of amplitude against EPSC rate within the red time window shown in *A* and *B*. *D*: plots of amplitude against EPSC rate for all recorded fibers of P20 pillar (green, *top left*), P20 modiolar (purple, *top middle*), P30 pillar (blue, *bottom left*), and P30 modiolar (orange, *bottom middle*) groups. On y-axis, amplitude was normalized with the amplitude at the response start for each synapse, so that all traces start at 1 on *y*-axis. On the *x*-axis, the EPSC rate was normalized with the maximal EPSC rate for each synapse, so that all traces conformed to the range of 0∼1. Thick traces are population average, while thin traces are individual synapses. At *right* is a replot of the population mean among the 4 groups for ease of direct comparison: 20 P, pillar at P20; 30 P, pillar at P30; 20 M, modiolar at P20; and 30 M, modiolar at P30. *E*: normalized amplitude at the maximal EPSC rate (normalized rate = 1) is compared among 4 groups as well as between pillar (black) vs. modiolar (gray) groups. Pillar synapses were statistically different than modiolar synapses (*P* = 0.0062). *F*: slope was obtained by linear regression on the steepest amplitude vs. rate plot as shown in *C* and compared among the 4 groups and between locations and again was different between pooled pillar and modiolar synapses (*P* = 0.0034). *Statistical significance for the compared measurements.

**Figure 8.**
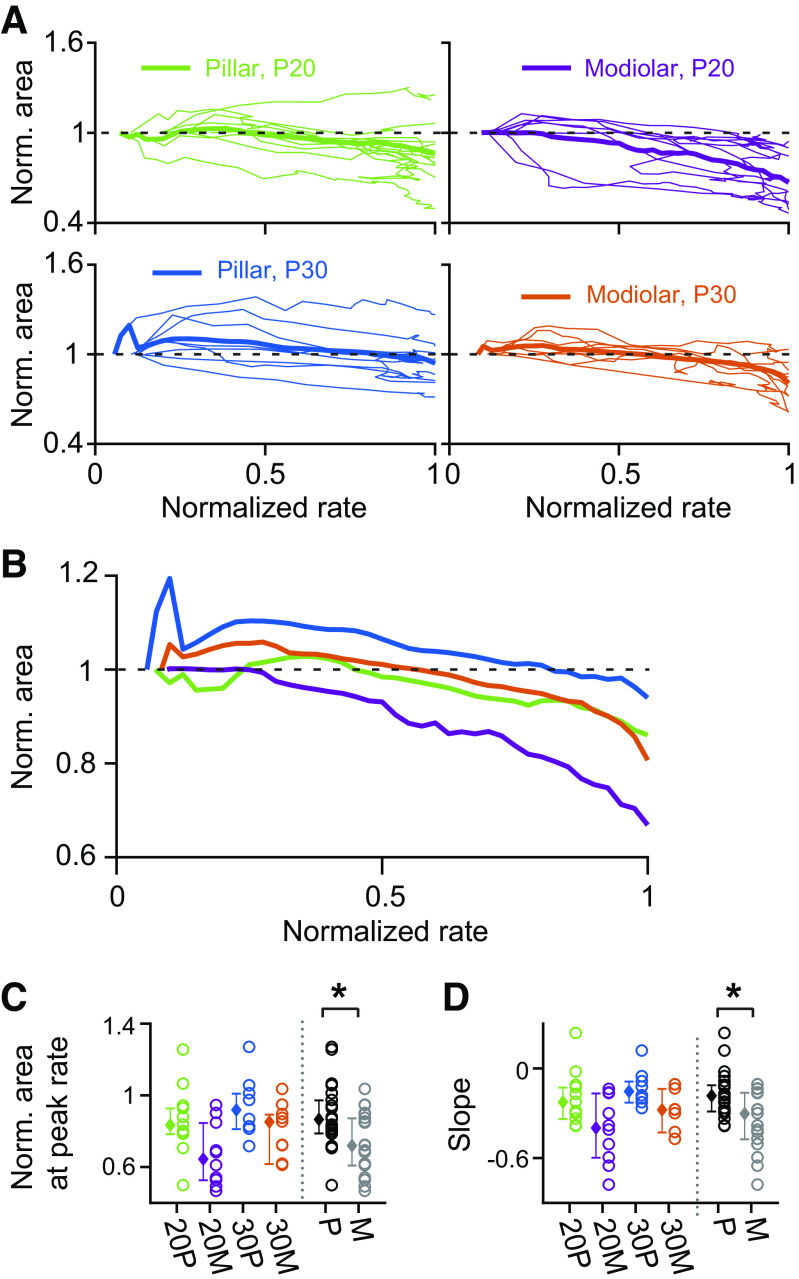
Excitatory postsynaptic currents (EPSC) area varies with increasing release rate. *A* and *B*: the dynamics of the EPSC area changes from the response start to the peak response for individual fibers at different ages and synaptic positions (*A*) and summarized (*B*) as the average response per group. *C*: EPSC area at the response start normalized to the area at maximal rate EPSC rate (normalized rate = 1) is compared among 4 groups as well as between pillar (black) vs. modiolar (gray) groups with pillar and modiolar synapses being statistically different (*P* = 0.0043): 20 P, pillar at postnatal day (P) 20; 30 P, pillar at P30; 20 M, modiolar at P20; and 30 M, modiolar at P30. *D*: same as *C* but for slope of area, with pillar being different from modiolar synapses (*P* = 0.0026). *Statistical significance for the compared measurements.

**Figure 9.**
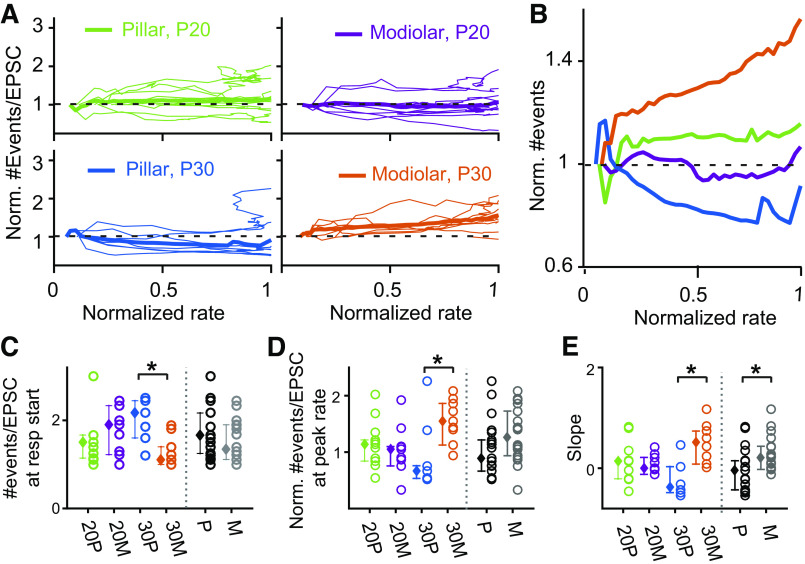
Variations in #events per excitatory postsynaptic current (EPSC) with increasing release rate. *A*: the dynamics of the number of events per EPSC (#E/EPSC) are presented for each synapse within each age and synaptic region from the start of the response to the peak response. *B*: the average of responses for each group is summarized. *C*: number of events per EPSC at the response start (the 1st-time bin that contained greater or equal to 5 EPSCs to reduce noise) is compared among 4 groups as well as between pillar (black) vs. modiolar (gray) groups. 20 P = pillar at postnatal day (P) 20, 30 P = pillar at P30, 20 M = modiolar at P20, and 30 M = modiolar at P30. *D*: summary of the #events/EPSC at the peak rate for each age and synaptic location as well as pooled across ages. *E*: steepest slope to the plots in *B*. *Statistical significance for the compared measurements.

Given the unexpected finding that the pillar and modiolar side fibers behaved similarly during prolonged stimulation, we hypothesized that perhaps differences were more dynamic and better observed during rate changes, rather than at steady state. Previous analysis may have masked these dynamic differences simply by the overwhelming proportion of EPSCs occurring during the steady-state time. We analyze time from onset until peak rate is achieved. This time period was selected because the inner hair cell membrane potential changes from a resting to a depolarized state, passing through the threshold potential at which release starts to occur. Thus this time window that spans a large rate change is of interest for finding differences in the behavior of pillar versus modiolar synapses. Additionally, since pillar and modiolar synapses are thought to have different thresholds for release that occurs at different hair cell membrane potentials, this time window may provide unique insight into the underlying differences between synapses (19). We examined how the EPSC amplitude, area, and number of events/EPSC changed over time from response onset to maximum rate and how these changes differ between age and fiber location (Figs. 7, 8, and 9). An example from a P36 modiolar fiber is shown in Fig. 7, *A–C*. Following 40-mM K^+^ application, its EPSC rate increased from 0 EPSCs/sec to a maximal EPSC rate of ∼60 EPSCs/s and then stabilized and sustained a rate above 30 EPSCs/sec for more than 2 min (Fig. 7*A*). The EPSC amplitude rapidly increased when K^+^ first started to elevate the rate, typically within 5 s (as shown in Figs. 4 and 5). At that point, while the rate was still increasing, the amplitude decreased over tens of seconds to a minimum just after the end of the rate increase, and gradually increased again (Fig. 7*B*). Figure 7*B* shows that for this modiolar fiber the amplitude reduced as the rate increased. In Fig. 7*C*, the time variable was eliminated by plotting EPSC amplitude against EPSC rate showing that amplitude decreases with increased rate.

In Fig. 7, *D–G*, data analysis focused on the time from the response onset to when the maximal EPSC rate is reached (as marked by the red box in Fig. 7, *A* and *B*). We characterized the relationship of EPSC amplitude and EPSC rate for all recorded fibers for the time between response onset and maximal EPSC rate, as we had done in Fig. 7*C* for the complete experiment. Individual fibers reached their maximal EPSC rate in response to 40-mM K^+^ application at different time points (ranged from 5 to 50 s, see Fig. 5*A*) so that taking the average of amplitude changes over time for a population of fibers could have obscured changes coupled directly to the EPSC release rate. We eliminated time as a variable from this analysis by plotting changes against the normalized rate (Fig. 7*D*).

The dynamics of the EPSC amplitude change with rate are variable among fibers. Figure 7*D* shows plots of EPSC amplitude versus rate from the response onset to the peak in rate for all recorded fibers. Here, the EPSC amplitude is normalized to the amplitude obtained from the first 100-ms bin that had at least five 5 EPSCs after the response onset. P30 Pillar synapses show an increased amplitude during the initial rate increase followed by a slower decline in amplitude. The initial increase in amplitude is less apparent at P20 pillar synapses or at either age for modiolar synapses. Most synapses, regardless of age or position show a decline in EPSC amplitude, as reflected in the decline of the population averages (bold lines in Fig. 7*D* and replotted in Fig. 7*E*). When comparing the EPSC amplitude versus rate relationship by age and location, we found that compared with pillar synapses, modiolar synapses had a greater decline in EPSC amplitude during the time the EPSC rate increases toward maximal. We statistically tested this using two measurements; *1*) normalized EPSC amplitude at the maximal EPSC rate, and *2*) the slope of a linear regression fit on the plot of EPSC amplitude versus EPSC rate. The normalized EPSC amplitudes at maximal EPSC rate were significantly smaller for modiolar synapses (*P* = 0.0062, Fig. 7*E*) and the slope of the EPSC amplitude decrease with rate was steeper for modiolar fibers (*P* = 0.0034, Fig. 7*F*; both by two-way ANOVA). In other words, pillar synapses sustain their EPSC amplitude significantly better than modiolar synapses, while the EPSC rate is increasing to peak. The EPSC amplitude decline could be due to the decrease in the number of available vesicles and in the amount of transmitter released per EPSCs and/or could be due to postsynaptic desensitization of glutamate receptors. If so, the EPSC amplitude decline might be larger for synapses with greater EPSC rates.

Next, we examined how EPSC area changes while EPSC rate is increasing toward the maximal EPSC rate (Fig. 8). The underlying hypothesis is that if the same amount of transmitter is being released, then the area will remain constant even when timing of release alters the maximal EPSC amplitude. Like in the analysis in Fig. 7*D*, the EPSC area was normalized to the area found at the response onset for each fiber and was plotted against the EPSC rate normalized to the maximal rate (Fig. 8, *A* and *B*). We found that modiolar synapses compared with pillar fibers show significantly greater decline in EPSC area during the time the EPSC rate is increasing toward the maximal EPSC rate (*P* = 0.0043 in Fig. 8*C* and *P* = 0.0026 in Fig. 8*D* by two-way ANOVA). This result is consistent with the finding that EPSC area has a positive, linear relationship with amplitude in a given synapse (data not shown): the larger the amplitude, the larger the area.

Lastly, we examined how the average number of events per EPSC (# events/EPSC) changes while the EPSC rate is increasing toward a maximal EPSC rate. Like the analysis of amplitude and area in Fig. 7*D* and Fig. 8*A*, the number of events per EPSC was normalized with the number of events per EPSC at the response onset and plotted against the EPSC rate normalized with the maximal EPSC rate. At P20, for both pillar and modiolar fibers, the number of events per EPSC does not on average change with rate (Fig. 9, *A* and *B*). Interestingly, a difference between locations appears at P30. The normalized number of events/EPSC at the maximal EPSC rate is significantly greater in P30 modiolar fibers than in P30 pillar synapses (*P* = 0.0194 with two-way ANOVA in Fig. 9*D*). Also, while the slopes of the rate versus #events/EPSC is flat at both positions for P20 fibers, the slope at P30 is positive for modiolar synapses and negative for pillar synapses (Fig. 9*B*). These data suggest that the pillar synapses are better able to generate large monophasic EPSCs when operating at high release rates while modiolar synapses are better at generating monophasic EPSCs when operating at lower release rates. In support of this conclusion, before the initial increase in amplitude in response to K^+^ application, P30 pillar fibers have a significantly greater number of events/EPSC than P30 modiolar fibers (Fig. 9*C*, *P* = 0.0483 by two-way ANOVA). Thus, if monophasic EPSCs are an indication of the operating range for the synapse, modiolar synapses are positioned to operate at lower stimulus levels.

### EPSC Properties Postpeak Rate

**Figure 10.**
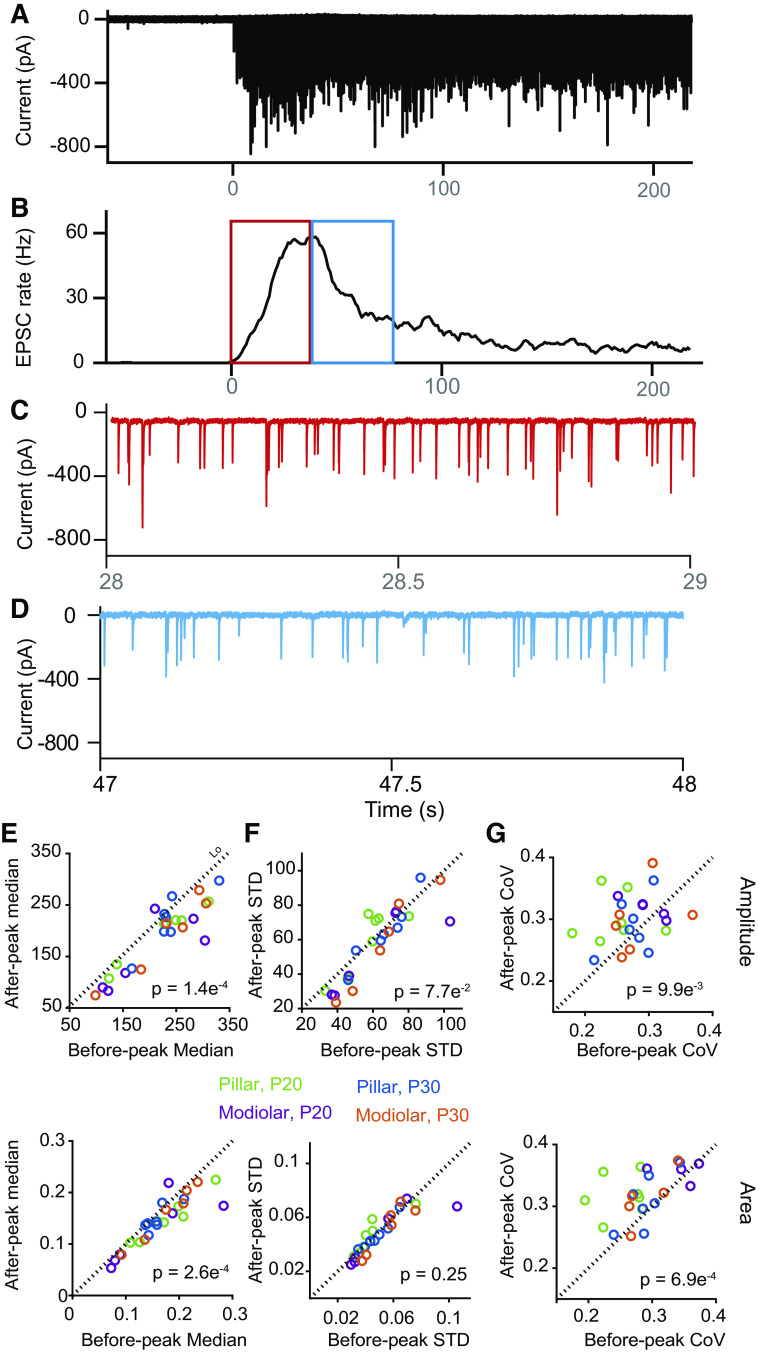
Excitatory postsynaptic current (EPSC) amplitude and area decrease as EPSC rate reduces post peak response. *A*: example of a postnatal day (P) 30 fiber’s response to K^+^ application. *B*: plot of the EPSC rate from *A*. The boxes indicate the time frames when the rates are rapidly increasing or decreasing. *C* and *D*: 1-s time window within the boxes in *B*. *E*: median amplitude (*top*) and area (*bottom*) for fibers in the 2 time windows showing that the after peak values are consistently lower than the pre peak values for both metrics regardless of age and synapse location (*P* values in *E*–*G*). *F*: illustration that despite the median values for amplitude and area decreasing the standard deviation (STD) does not change and thus the coefficient of variation (CoV) change can be explained. *G*: demonstration that CoV for amplitude (*top*) and area (*bottom*) is greater for EPSCs during the falling phase of the rate as compared with the rising phase. Green represents pillar side at P20, purple modiolar side at P20, blue pillar side at P30, and orange modiolar side at P30.

Figures 7, 8, and 9 demonstrate that EPSC properties change dynamically as a function of rate and that these differences separate by synapse location. Most synapses show a significant decrease in rate following peak rate. We compared EPSC amplitude and area prepeak and postpeak over a comparable time frame to further investigate the dynamics associated with release. An example recording and EPSC rate plot are presented in Fig. 10, *A* and *B*. The time windows analyzed are presented in the boxes in Fig. 10*B* with an expanded view of the EPSCs within the time window presented in Fig. 10*C* for prepeak and Fig. 10*D* for postpeak. A reduced amplitude is apparent in the EPSCs postpeak in this example. We quantified EPSC properties during these two time periods by first comparing the median amplitude and area before and after the peak rate was achieved. Before peak rate amplitudes and areas (Fig. 10*E*, *P* = 0.00014 and 0.00026, respectively) were consistently larger than postpeak rate amplitudes and areas. These plots depict the median amplitude or area for each recorded fiber for each age and synapse location (color coded). Although both amplitude and area were significantly different pre- and postpeak rate, there was not a difference in the standard deviation of each measurement (Fig. 10*F*, *P* = 0.077 and 0.25, respectively). A consequence of the median changing but not the variance is that the coefficient of variation (CoV) for amplitude and area of the EPSCs was increased during postpeak responses (Fig. 10*G*, *P* = 0.0099 and 0.00069, respectively). This finding is surprising and different than what was found for the increasing rate time period where a change in events per EPSC is predicted to have a standard deviation scaling with the mean and therefore no effect on CoV. Here we postulate a postsynaptic effect, likely desensitization reducing the mean amplitude/area but having no effect on release mechanisms that drive the variance.

### Initial Application of 40 mM K^+^ Dramatically Changes EPSC Properties

To this point we have investigated effects on EPSC size and frequency and their dependence on age and synapse location. Our final investigation is of the underlying substructure of the EPSC as identified via deconvolution as a means of identifying changes in release properties between age and synapse location. The analysis is not dependent on a knowledge of the underlying mechanisms associated with generating the broad range of EPSCs as defined by size and shape. However, the analysis does allow for hypotheses regarding these mechanisms to be derived.

To investigate the effects of hair cell depolarization on EPSC properties, the immediate effects of 40-mM K^+^ application at the response onset were analyzed. Response onset is relative to the change in EPSC frequency because of the variability in onset of effect likely due to the diffusion barriers associated with 40 mM K^+^ reaching the basolateral surfaces of the IHCs. Also note, that in this experimental setting, before 40-mM K^+^ application, IHCs membrane potentials were at negative levels, at approximately −65 mV so the rate of EPSCs was very low (23). At these negative resting potentials release is limited (31). Thus this analysis is biophysical in nature and does not address the physiological nature of the recorded EPSCs.

**Figure 11.**
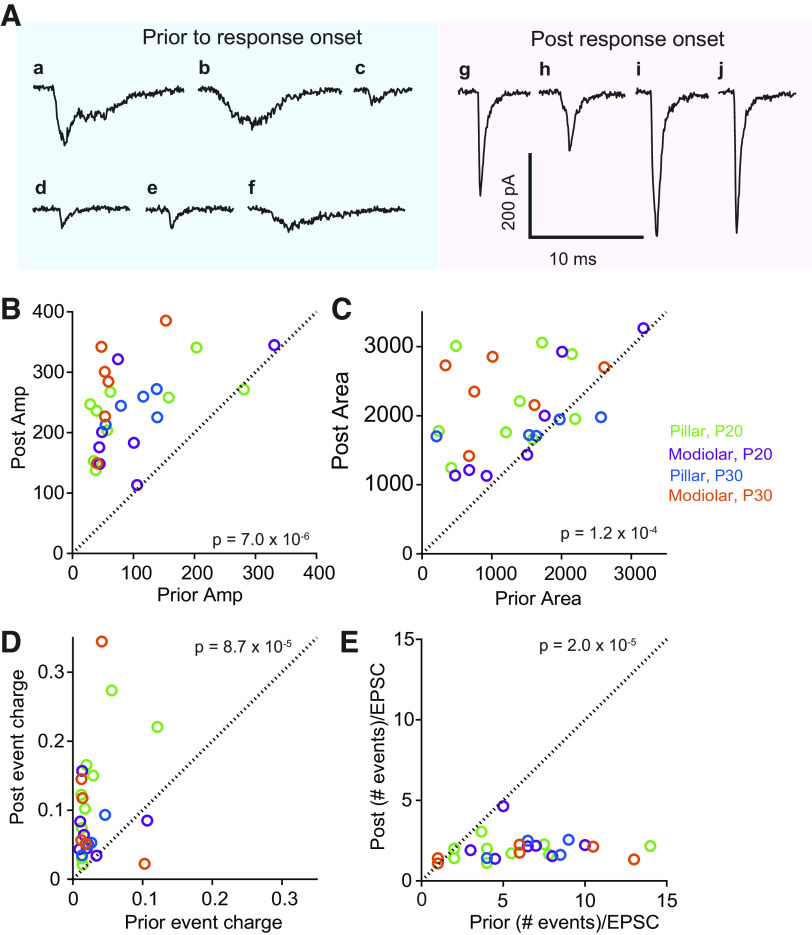
Excitatory postsynaptic currents (EPSCs) vary in amplitude and kinetics depending on release rate. *A*: exemplary EPSCs found before the onset of response to 40-mM K^+^ application (*a*–*f*) and postonset (*g*–*j*). All EPSC examples except *d* and *e* came from a single pillar synapse. *B*: the mean EPSC amplitude before K^+^ application is plotted against the post application mean showing a strong increase in amplitude during stimulation. *C*: similar plot of the EPSC area prior and poststimulation, again demonstrating an increased EPSC area with stimulation. P, postnatal day. *D*: decrease in number of events per EPSC, as determined from deconvolution. *E*: increase in charge per event during stimulation as compared with prestimulation. No difference was found between age or synapse location.

Representative EPSC waveforms found before and immediately after the onset of the response to the 40-mM K^+^ application are shown in Fig. 11*A*. The postresponse onset data included EPSCs from 0–5 s to examine the most immediate effect of 40 mM K^+^ (also see Fig. 4, *insets*). Before the response onset, EPSCs tend to be small and slow, with multiphasic or monophasic waveforms (Fig. 11*A, a–f*), while after the response onset typically larger EPSCs with faster rise times and exponential decays appear (Fig. 11*A*, *g–j*). Both types of EPSCs occur throughout the recording period. EPSC amplitudes become significantly larger with the 40-mM K^+^ application (Fig. 11*B*, *P* = 7.0 × 10^−6^ by rank-sum test) when compared with before the response onset. The plot in Fig. 11*B* illustrates for each fiber recording the relationship of median EPSC amplitude before and during K^+^ application where all but 3 fibers show an increase in amplitude following K^+^ application. No difference was found between age or bouton position. Similar to the amplitude, EPSC area is significantly larger after the response onset (Fig. 11*C*, *P* = 1.2 × 10^−4^). The charge of individual events within the EPSC, which is equivalent to gain times the kernel (see Fig. 2) is increased when activity is increased (Fig. 11*D*, *P* = 1.78 × 10^−6^). The number of events per EPSC is significantly larger for EPSCs occurring before the response onset compared with those postonset (Fig. 11*E*, *P* = 2.0 × 10^−5^). These data show that increased activity initially results in EPSCs with larger amplitudes and areas and smaller widths, with a decrease in the number of events/EPSC, while the size of the individual events within EPSCs increases. Without imposing a particular release mechanism, one interpretation of these data is that the underlying release process is consolidated into a more uniform output.

### With Increased Number of Events/EPSC, EPSC Amplitude Decreases

A prediction from the above data is that there would be a negative correlation between number of events per EPSC and EPSC amplitude. We investigated this relationship across the entire duration of the recording for each age and location. Overlap of release events from independent release sites (here called “doubles”) might impact this analysis, as such overlapping events will be mistakenly recognized as multiphasic events. However, such doubles can be discriminated from multiphasic events by their increased area. We developed a method to remove doubles (see Fig. 3) and present data in Fig. 12 after doubles have been removed. We find that the EPSC amplitude is inversely related to the number of events per EPSC for all four age/location groups [Fig. 12*A*; Refs. 27, 32; see Young et al. (28)]. We fit a line to the steepest portion of the plots in Fig. 12*A* and present the resultant slopes in Fig. 12*B*. For both amplitude and area, we found little decrease between 1 and 2 #events/EPSC and so fit the data between 2 and 4+ events for both measurements. We found no difference between negative slope values for age or synapse location. We similarly examined the relationship between the EPSC area and number of events/EPSC (Fig. 12*C*). Surprisingly, in contrast to previous reports that found a close to constant EPSC area for different number of events/EPSC [Refs. 27, 32; see Young et al. (28)], we found near-zero and slightly decreasing areas as the number of events/EPSC increased (negative slopes, defined by fitting the steepest portion of the plots from Fig. 12*C*) for the area plots (Fig. 12*D*). The slope is significantly negative when pooled across all 4 groups (mean = −27.0 ± 24.1, *P* = 3.99 × 10^−8^, Fig. 12*D*), as well as for individually tested groups. The magnitude of the slope is different between the pillar and modiolar synapses, greater for the modiolar when pooled (*P* = 0.045). Potentially the higher release rate results in more desensitization that manifests itself more over the time course of the multievent EPSCs.

### Two Populations of Events Contribute to Events/EPSC

**Figure 13.**
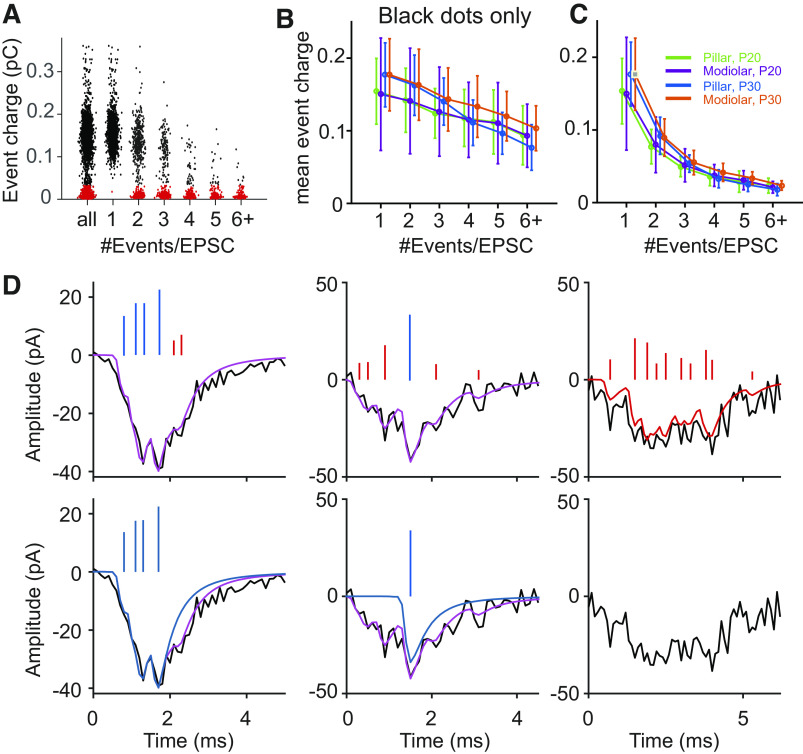
Two populations of events are identified based on charge where one population reduces in amplitude and number with #events/excitatory postsynaptic current (EPSC) and the other increases in number and remains constant in amplitude with #events/EPSC. *A*: charge (gain times the kernel, (see Fig. 2) of deconvolved events as a function of #events/EPSC for a pillar synapse at postnatal day (P) P36. Each dot denotes one event and is placed on the *x*-axis at the total number of events in the EPSC to which the event belongs. For example, if an EPSC is made of 3 events with charges of 0.5 pC, 0.2 pC, and 0.05 pC, then these 3 charges will be plotted at the #events/EPSC of 3 at the *x*-axis. For “all” (the *leftmost*) column, all events aggregated across #events/EPSC are plotted. The events are colored in black and red, showing the result of clustering by K-means (K = 2). The distribution of event charges aggregated across all #events/EPSC was used for clustering. *B*: Population-averaged mean event charge is plotted as a function of # events/EPSC for the events denoted as black dots in *A*. *C*: population-averaged mean event charge is plotted as a function of # events/EPSC for combined black and red populations. Error bars are SD. *D*: examples of 3 EPSC waveforms from the same recording as in *A*. The *top row* of EPSCs, shown as the black traces, is comprised of a mix of red and black events. The blue and red lines above the traces represent individual events where the height is the charge of that event and the position represents when it occurred, and the red are from the red cluster and blue from the black cluster in *A*. The purple trace shows the fit of the deconvolution result which sums the red and blue events. The *leftmost* EPSC has a majority of blue events, while the mid has a majority of red events and the right hand EPSC only contains red events; thus the simulated EPSC is red. The *bottom traces* show the predicted EPSCs if the red events were excluded. Here the traces are blue because they only represent blue events. The *rightmost* EPSC is excluded because there are no blue events. Simulations show poor reproduction of EPSCs if red events are excluded.

To this point, the unstated underlying assumption is that there is a common mechanism for multiphasic and monophasic events and that the underlying release mechanisms dictate the proportion of each component found during stimulation. To test this assumption, we evaluated this distribution of both event types, Additionally, the variance in the data for either amplitude or area of events/EPSC is unusually high, prompting us to further explore these distributions. To this end, we examined the charge of individual events, which is defined as the gain times the kernel shape (Fig. 2). We aimed to directly test the hypothesis that individual events are smaller in EPSCs having a larger number of events. An example from a single synapse is presented in Fig. 13*A* showing the charge of individual events plotted against the number of events/EPSC. Notice that there are two clusters in the distributions. This clustering was present in all recorded single synapses. We applied an unsupervised clustering technique, K-means, and divided all events (the Fig. 13*A*, *leftmost column*) into two groups denoted in black and red. The result of clustering was reflected on the subgroups of EPSCs with 1–6+ number of events/EPSC. The black distribution has the larger charge/EPSC and contributes substantially more to the distributions with the smaller number of events/EPSC while the red distribution predominates in the EPSCs with a larger number of events. The cluster represented by the black dots decreases in charge as the #events/EPSC increases both for the individual synapse and the population of synapses (Fig. 13, *A* and *B*, respectively). The population of events represented by the red dots shows no change or a slight increase in charge as the #events/EPSC is increased (data not shown). Thus the overall distribution of mean event charge decreases dramatically due to both the increased contribution of the red events and the decrease in charge of the black events (Fig. 13*C*). No differences were observed with either age or synapse location for these populations of events.

Interpretation of the red versus black clusters is confounded by the likely artifact imposed by the deconvolution algorithm of overfitting, where despite the high penalty that accompanies the addition of events, there remains some small level of “extra” events. In our approach this error is likely low due to the conservative approach used by our algorithm, where potentially we might actually underestimate red events. Examples of EPSCs are presented in Fig. 11*D* to demonstrate the likely necessity of red events in reproducing EPSCs. Figure 11*D*, *top*, shows three complex EPSCs where the leftmost is comprised almost entirely of black (shown as blue) events, the middle comprises a majority of red events and the right of only red events. The events are depicted in time by the location of the line above the EPSC (black trace) and the charge indicated by the height of the line, with the color denoting population. The purple traces depict a fit with the sum of the red and blue populations, blue represents a fit with only blue and red only red events. It is clear from the smooth traces produced by the deconvolution that the algorithm is not overproducing red events. The *bottom* traces show the deconvolution output without red events where the EPSCs are not reproduced in time (blue traces) and the *rightmost* EPSC is completely missed because it is composed of only red events. More experiments are needed to investigate the properties and mechanisms associated with generating these independent population of small events, but the data clearly demonstrate that both populations are necessary.

## DISCUSSION

Present work investigated differences in hair cell afferent fiber synapses based on age (P20 and P30) and synaptic location around the inner hair cell (pillar and modiolar). We investigated EPSC size, shape, and rate in response to an elevated extracellular K^+^ that increased release probability. We investigated both steady-state effects on EPSCs as well as changes in properties associated with dynamic increases or decreases in EPSC rates. Finally, we investigated the underlying composition of individual EPSCs. The analyses above were done with respect to age and synapse location.

### Pillar Synapses Mature Later Than Modiolar Synapses

The maximal EPSC rate increased for pillar synapses between P20 and P30, while modiolar fibers remained constant, suggesting a later maturation of pillar synapses (Fig. 5). The change in maximum rate with age suggests a presynaptic mechanism that may be related to the availability of releasable vesicles or potentially a change in number of release sites. This is consistent with the finding by Ref. 22, showing the percentage of high spontaneous rate fibers (presumed features of pillar fibers) increasing between P20 and P30. Morphological maturation of the synaptic ribbon, an electron dense structure found at the presynaptic terminals in IHCs is not complete until P28; the gradients between P14 and P21 are reversed from what is found in adult at most frequency regions from P14 to P21 (20). Thus the morphological and electrophysiological timing of maturation are in accord. However, we do not have a functional correlate to changes in ribbon size or postsynaptic glutamate receptor plaque, although we speculate about how changes in calcium channel distributions may underlie some of the responses measured.

Pillar synapses also show an increase in median EPSC amplitude and area between P20 and P30 (Fig. 6), further supporting the conclusion of later maturation for pillar synapses. At P20 or P30, there were no detected difference with synapse location for amplitude or area when averaging over the entire recording period despite there being a difference morphologically in the size of the postsynaptic receptor density plaque. Although an overlap between physiological maturation and morphological maturation exists, one does not appear to account for the other. Specifically, the postsynaptic change in receptor plaque size does not explain the amplitude increase. Although it did not reach statistical significance, there was a trend for events/EPSC to reduce for pillar synapses, which does correlate with an increased amplitude and might underlie the age-dependent change in amplitude. Taken in total, the functional maturation appears to be presynaptic in origin.

### Pillar and Modiolar Synapses Show No Major Differences Over the Entire Recording Time Course

Comparisons of rate, amplitude and area as well as events/EPSC for pillar and modiolar synapses found no major differences. These comparisons were performed in the presence of 40 mM K^+^, which depolarizes hair cells to approximately −20 mV (23) and so are considered at high synapse activity. It was surprising that no functional correlates were observed for the morphological differences such as ribbon size and glutamate receptor plaque size; however, it is not unprecedented, given the limited changes in synaptic properties associated with the loss of CTBP2 (23, 33). Thus the differences between synapses are likely more subtle than this general analysis can identify or perhaps the differences in SGN properties are more associated with postsynaptic excitation properties. Recent data from both frog and mammal suggests that postsynaptic fibers vary in fundamental properties like input resistance, membrane potential and spike threshold; additional (21, 34) investigations are needed to evaluate excitability based on synapse location.

### Release Properties Change as Release Rates Increase

We hypothesized that synapses might be specialized to respond to changes in stimulation differently and that the analysis of steady-state high stimulus responses might mask synaptic differences related to rate changes. Thus we selected the specific time frames when EPSC rates were increasing or decreasing for comparison. We analyzed EPSC properties during the K^+^-induced increase in rate, until achieving the maximal release rate (Figs. 7, 8, and 9). At P30, EPSC amplitude decreased more over this time period in modiolar synapses. The area of the EPSCs also decreased more in modiolar fibers suggesting a reduction in total release. Pillar fibers showed an increase/maintenance in amplitude and area over the same time period, with a decrease in event number per EPSC. Together these data suggest that pillar fibers are better capable of maintaining large amplitude, monophasic EPSCs at times of large stimulation. Modiolar synapses show larger monophasic EPSCs at the onset of the stimulus and this degrades to multiphasic as the rate continues to increase, thus suggesting these fibers are better capable of maintaining monophasic EPSCs at lower stimulus intensities.

### How Is Synaptic Specialization Obtained?

A computational model described how the synchrony of elementary events might be affected by Ca^2+^ channel density and open time (32) and suggested that monophasic EPSCs might be triggered by high Ca^2+^ concentration and short Ca^2+^ channel mean open times, whereas multiphasic EPSCs might be triggered by lower Ca^2+^ concentration and longer open times. According to this model, our result of a lower number of events/EPSC at modiolar fibers at the start of the response can be explained with a larger intracellular Ca^2+^ concentration in response to depolarization at modiolar compared with pillar synapses. This is consistent with previous findings that the number of Ca^2+^ channels (inferred by immunohistochemistry) and Ca^2+^ current influx (revealed by optical imaging of Ca^2+^ indicator) are larger on the modiolar side of presynaptic terminals and that calcium channels activate at more hyperpolarized potentials as compared with the pillar side (19, 35). Potentially the calcium differences at synapses underlies the late changes in ribbon size, similar to that reported in zebrafish (36).

We found that the modiolar synapses were less capable of generating monophasic EPSCs during stimulations evoking higher release rates. Although not directly assessed in the calcium model, we hypothesize that the larger calcium load likely saturates clearance mechanisms, which can serve to decouple release mechanisms. Additionally, calcium clearance mechanisms might differ between synapse locations as well and warrants further investigation. The more efficient coupling of release at pillar synapses may be due to the lower channel density allowing clearance to be maintained. To summarize, more calcium channels that activate at a hyperpolarized potential for modiolar fibers may well maintain release for smaller stimulations but result in a larger calcium load at larger stimulations that could overwhelm the buffering capacity of the presynapse, whereas the opposite is true for pillar synapses.

### Functional Relevance of Pillar and Modiolar Synapse Specializations

Data presented here suggest that the main difference between pillar and modiolar synapses lies in the level of ability to generate large monophasic EPSCs at different stimulus intensities. Modiolar synapses are specialized for lower stimulus intensities and pillar for higher stimulus levels. Although all synapses appear to have similar release properties and the ability to coordinate release properties, we are suggesting that differences in calcium homeostasis put these synapses at different operating points as relates to generating synchronous (large amplitude single peaked) EPSCs.

If we assume that (for similar EPSC areas) monophasic EPSCs have a higher probability of triggering action potentials and of doing this with reduced variance in timing, then stimulus-dependent differences coupled with postsynaptic excitability likely underlie differences in spontaneous activity, dynamic range and spike timing between pillar and modiolar fibers. However, neural desynchronization may also be important. Cerebellar Purkinje cells suggest that desynchronization of EPSCs enhanced axonal propagation of individual spikelets to target cells, leading to more efficient information transfer (37). Thus more direct evidence by investigating postprocessing of synaptic inputs is needed to be sure of the role of release synchronization. Regardless of the physiological role, this property is critical to signal processing differences between these synapses. Single unit recordings demonstrate that SR of auditory nerve fibers not only correlate with intensity threshold but also with other parameters including response latency, the size of onset response, the speed of adaptation, resistance to masking noise, and phase-locking ability (8, 13, 14). Mechanisms underlying these differential properties will need to be further investigated in the future. Likely, presynaptic specializations can only account for part of the response properties measured in vivo. Postsynaptic specializations need further quantification to demonstrate the key properties of EPSCs needed to generate sustained spiking (34) (see Ref. 21, #530).

### Stimulus Dependent Intra-EPSC Changes

EPSC amplitude and area increased almost immediately upon exposure to elevated external K^+^ (Fig. 11), with a corresponding decrease in #events per EPSC and an increase in the charge per event within the EPSC. This observation was most prevalent for pillar synapses. Our observations in part reflect hair cell resting potentials being hyperpolarized before stimulation so that release rates are very low. These data are in agreement with data from lower frequency hearing organs like turtle and frog where experiments that reduced release probability, like hair cell hyperpolarization or increased intracellular calcium buffering, reduced EPSC amplitudes (38–40). In mammalian systems, because of the single synapse per fiber, when release probability is driven to be very close to zero, the window for detecting a reduction in amplitude is limited (i.e., there are very few if any events at the one synapse). The amplitude being sensitive to activity is consistent with calcium homeostasis regulating the timing of acquisition of mature release properties as previously described (30).

### Multivesicular Versus Uniquantal Models of Synaptic Vesicle Release

The EPSCs recorded at the hair cell afferent fiber synapse are unique in being quite large, often quite variable in amplitude but having similar onset and decay times despite differences in amplitude (41). The multivesicular release (MVR) model of release proposes multiple synaptic vesicles undergo co-operative, synchronous fusion, enabling EPSCs with a wide range of EPSC amplitude and similar kinetics (27, 41–43). Synchrony, in this context refers to the time of release between vesicles. Data also exist from afferent recordings in frog and turtle to support MVR (38, 44). Changes in timing of fusion of these vesicles could account for the properties presented here. For example, the synchronization of vesicle release could underlie the increased amplitude, narrowing of the width, and reduced number of events per EPSC observed in pillar fibers as release rate increases. Events per EPSC could likely reflect timing of release between independent vesicles so that as the time between fusion events is reduced the number of events is reduced and the charge per event is increased. In contrast, the underlying elementary event can also be interpreted as flickers of the fusion pore that vary in open time until complete fusion occurs in a kiss and run model of uniquantal release (UQR) (32). This model proposes the existence of dynamic fusion pore openings (flickers). UQR could similarly explain the initial amount of release simply by increasing the open time of the fusion pore (32). An increased open time would result in larger amplitude EPSCs with fewer events and greater charge. If pore open times inversely correlate with number of flickers, then data presented here can also be reproduced. The deconvolution method used here assumes that the EPSC is created by a linear summation of discrete, elementary events, but we have not assigned a molecular definition to these events. The elementary events in our deconvolution model could be interpreted as either a fusion pore opening or as release events of independent vesicles.

The deconvolution analysis provides interesting insight into the makeup of the complex EPSCs having more than one event per EPSC. These EPSCs have a broad distribution of small event charges (Fig. 13*A*, red events) that do not create a classical Gaussian distribution of unitary size. The presence of these events was consistent across age and synapse location but was observed more often when release was at a lower rate. A similar population of small EPSCs was described in the ribeye knockout mutant (23). Although our data and analysis cannot definitively identify a mechanism, they do argue that conventional vesicle fusion is unlikely to explain this population of events as there is not a well-defined minimal size. Additionally, if we assume classical quantal behavior, meaning that the red dots represent the distribution of unitary events and that the black dots represent a summation of multiple red dots, simply dividing the mean event charge (Fig. 13*A*, black dots) by the mean event charge (red dots) estimates a number of 16. The idea of 16 vesicles simultaneously fusing to provide the larger EPSCs, without altering kinetics seems unlikely and so our data does not cleanly distinguish between UQR from MVR.

One hypothesis generated by considering the present data is that the energy for vesicle fusion is greatly reduced at these synapses due to the specialized synaptic molecules dictating release (45). The reduced energy potentiates high fusion rates and rapid fusion events. A consequence of this specialization is creating an environment where there is some low level of UQR when outside of physiological calcium ranges, for example, when cells are hyperpolarized prestimulation. Thus the red population of events perhaps represent UQR and are a consequence of this reduced fusion energy while the black population represent full fusion events and likely MVR. Under physiological conditions we expect the black population of events to predominate. Rather than UVR and MVR being mutually exclusive, we are suggesting that both are likely relevant depending on stimulus intensity.

## GRANTS

This work was supported by National Institute on Deafness and Other Communication Disorders (NIDCD) R01-009913 (to A.J.R.), R01-DC-006476 (to E.G.), and F32-DC-013721 (to M.N.). NIDCD Grant P30-DC-005211 to the Center for Hearing and Balance at Johns Hopkins, the David M. Rubenstein Fund for Hearing Research, and the John Mitchell, Jr. Trust (to E.G.). We are grateful to the Oberndorf family and other Stanford Initiative to Cure Hearing Loss contributors for support.

## DISCLOSURES

No conflicts of interest, financial or otherwise, are declared by the authors.

## AUTHOR CONTRIBUTIONS

M.N. and A.J.R. conceived and designed research; M.N. performed experiments; M.N. and E.D.Y. analyzed data; M.N., E.D.Y., E.G., and A.J.R. interpreted results of experiments; M.N. and A.J.R. prepared figures; M.N. and A.J.R. drafted manuscript; M.N., E.D.Y., E.G., and A.J.R. edited and revised manuscript; M.N., E.D.Y., E.G., and A.J.R. approved final version of manuscript.
